# “On Suggestion” by John Bostock, 1923: A comparison with twenty-first century understandings of the placebo effect

**DOI:** 10.1177/10398562231187982

**Published:** 2023-07-04

**Authors:** Alison Clayton

**Affiliations:** School of Historical and Philosophical Studies, 153396 University of Melbourne, Melbourne, Australia

**Keywords:** history of psychiatry, suggestion, placebo effect, Australian psychiatry, John Bostock

## Abstract

**Objectives:**

This paper describes Australian psychiatrist John Bostock’s 1923 concept of suggestion and compares it to our understandings, in 2023, of the placebo effect.

**Conclusions:**

Bostock’s 1923 article on “suggestion” gives us a glimpse of the history of Australian psychiatry. It also stimulates thought about the current understandings of the placebo effect. Now, as then, placebo effects can play a critical role in patient outcomes. However, careful consideration is required to ensure contemporary ethical standards are met and harm is not done.

A century ago, Australian psychiatrist John Bostock (1892–1987) published his paper, “On Suggestion, with Examples taken from Mental Hospital Practice.”^1^ Bostock, a Glasgow born and recently emigrated London medical graduate, worked as a medical officer at Western Australia’s Claremont Hospital for the Insane. He went on to practice psychiatry in Sydney and Brisbane and was, in 1938, a founding member of the Royal Australian College of Physicians. In 1948, he served as President of the Australasian Association of Psychiatry.^2^ My brief article describes the key points of Bostock’s description of suggestion. His description of “therapeutic suggestion” is then compared to present-day understandings of the placebo effect. I conclude with some reflections on the ongoing clinical and research implications of the placebo effect.

Drawing on the work of British psychologist William McDougall, Bostock described suggestion as occurring ubiquitously in ordinary life and being “a process of communication resulting in the acceptance with conviction of the communicated proposition in the absence of logically adequate grounds for its acceptance.” Bostock postulated an evolutionary perspective, describing suggestion as “an ancient means of communication” that did not necessarily require spoken language. He emphasized that no human, however learned, was immune from suggestibility.

Bostock considered suggestion to be “one of the doctor’s best tried, most trusted, oldest, most ubiquitous and retiring of kind helpers.” He described that the optimistic surgeon, promising recovery after an appendectomy, owed “not a little to the effect of his cheery words on a susceptible patient.” Likewise, physicians’ medicines “would have but little effect without the verbal pronouncement that his subject will feel better afterwards.” Bostock pointed to the history of medicine that was replete with “remedies” which had had their “vogue” and then fallen into “oblivion.” Anton Mesmer’s magnetic fluid theory and mesmerism was mentioned as an example. The common ingredient, Bostock argued, was suggestion—the patient was told he would be cured, expected to be cured, and was thereby cured.

Bostock listed several factors that could increase the power of suggestion. First, the subject’s lack of knowledge of the issue increased their vulnerability. This accounted for the increased gullibility found in children or in patients with neuropathological conditions, such as general paralysis of the insane or Korsakoff’s syndrome. Second, a prestigious or charismatic individual had enhanced suggestive powers. Bostock described this as “prestige suggestion,” and thought that a “forceful and impressive physician should have, other things being equal, better results than his diffident colleagues.” Third, a subject’s suggestibility could be increased by temporary brain states, for example, induced by fatigue, hypnotism, or crowd psychology. Bostock described that one may enter a crowd “cool and collected” and one may leave it “with heightened emotion, thanks to the contagiousness of sympathy, imitation and suggestion.”

Bostock expanded on possible mechanisms that might underpin crowd psychology. He discussed the “herd instinct,” and made special mention of the impact of war in inciting crowd behavior. In addition to McDougall, it is likely that Bostock was acquainted with Wilfred Trotter, a leading British surgeon, and his book, *Instincts of the Herd in Peace and War*.^3^ In Trotter’s view, suggestibility did not indicate a preference for the irrational; rather it indicated the importance of herd opinion, rational or not, to an individual’s psychology. All three men were at the University of London prior to World War I. Bostock graduated in 1914 and then served as a surgeon in the war, including at Gallipoli.^2^ In his paper, Bostock also described the impact psychology could have on physiology. He described that fear was associated with racing heart, dilated pupils, micturition, defecation and sweaty palms, and that bad news, such as in a telegram, may cause syncope, or in a pregnant woman a miscarriage. It seems likely that Bostock was here drawing on his wartime experiences.

Bostock provided several fascinating clinical vignettes of patients whom he treated by therapeutic suggestion. He practiced a wide variety of therapeutic suggestive methods, including simple firm instruction, the laying on of hands, placebo medication and sham operations. For the last, he described the following. The patient was a young woman with psychotic symptoms, including the belief that the spirits were talking to her through a hole in her head. The doctor and nurses informed her that a brain operation to close this hole would resolve her problems. She was taken to the operating theater, had her hair cut, alcohol and ethyl chloride were applied, and the area pricked with a needle. A dressing of “unguentum hydragyrum” (a mercurial compound) and a bandage completed the “operation.” Bostock reported that the patient’s “voices disappeared immediately” and there was considerable improvement in her general behavior. It is of interest for us to note that this sham brain operation took place 15 years before the introduction of the lobotomy, which, without any placebo trials, became a popular and widely used, but later discredited, psychiatric treatment.^4^ Bostock’s conclusions about suggestion as a treatment method were measured. He noted his case series was too small, and it was too early to draw conclusions, but that the results were encouraging, and further trials were warranted.^1^

Bostock’s article holds more importance than just being a curiosity from Australian psychiatry’s history. It acts as a reminder that therapeutic suggestion, or what we now mostly call placebo effects,^5^ are part of the warp and weft of the therapeutic relationships and can be critically important to patient outcomes.^6^ As defined in a recent consensus statement,^7^ placebo (beneficial) and nocebo (deleterious) effects occur in clinical or research contexts and are due to psychobiological mechanisms evoked by the context, especially patients’ expectations, rather than any specific effect of the intervention. Placebo effects are viewed as not only occurring with the prescription of placebo (inert) pills, but as substantially impacting on the efficacy and side effects of active medical treatments. Furthermore, placebo effects can occur with the use of open label, not just hidden (deceptive), placebos. The specific condition is also relevant, for example, robust evidence supports placebo effects as being significant in a wide range of conditions including pain, depression, fatigue, and Parkinson’s disease.^7^ However, although placebos can impact on symptoms of cancer, there is no evidence they can “shrink tumors.”^8^

There have been significant developments in our understandings of the placebo effect’s physiological concomitants.^5,9-11^ Complex neurobiological mechanisms are implicated, including release of neurotransmitters (e.g., endorphins, endocannabinoids, oxytocin, and dopamine) and activation of specific areas of the brain (e.g., prefrontal cortex and amygdala). These changes are associated with an increase sense of well-being, and impact on cardiovascular, respiratory, immune, and endocrine function, which may contribute to a patient’s clinical improvement.^11^ Like Bostock, contemporary placebo researchers note the important role played by the clinician’s personality, social learning, and social contagion—especially, nowadays, as induced via social media.^9,10^ For example, currently, the role of social media platforms acting as “spread vectors” for mental illness symptoms is receiving scrutiny; as seen in the phenomenon of “TikTok’s sick role subculture” and the increasing numbers of youth presenting to clinics with functional tic-like behaviors and dissociative identity disorders.^12^

Some contemporary placebo researchers, like Bostock speculated for suggestion, provide an evolutionary perspective on the placebo effect. For example, theorizing that an individual’s trust in a society’s healer (whether doctor, shaman, or wise woman) and the placebo response may improve survival and provide an evolutionary advantage.^9^ One critical difference between Bostock’s account of therapeutic suggestion and contemporary discussions of placebo effect is the attention given to ethical concerns. Bostock does not mention any ethical concerns regarding the use sham treatments and no mention is made of patient consent. In contrast, the contemporary placebo literature details complex clinical and research ethical issues.^7-9^

It is important to acknowledge that, despite this being the era of evidence-based medicine and double-blind randomized controlled trials (DBRCT), there is still the risk that contemporary treatments will, in later times, be discovered to not have specific treatment effects. First, because some 21st century treatments are not underpinned by DBRCT (sometimes the nature of condition or treatment precludes this possibility, sometimes researchers refuse to undertake DBRCT or claim such trials are unethical).^4^ Second, because, DBRCT maybe poorly designed and “breaking blind” can occur, especially with treatments that have notable and known side effects, as is the case for many psychotropic medications.^13-15^ This, thus, compromises such studies’ ability to distinguish placebo effect from specific treatment effect.

In contemporary clinical practice, the placebo effect is best considered as a double-edged sword. A clinical setting in which an empathic clinician listens to the patient, is supportive and encouraging, while giving truthful information, creates a legitimate “therapeutic bias” by giving the patient hope and expectation of improvement.^16^ It is good clinical practice for clinicians to use these methods to harness placebo effects and improve patient outcomes.^6,8^ However, when treatments lack evidence of efficacy, relying on placebo effects to improve outcomes, especially without open disclosure to the patient, is problematic and not consistent with informed consent requirements or international consensus guidelines.^7^ Furthermore, the use of active placebos, with potential for adverse effects, may lead patients to feel not only that they have been deceived, but that they have also been unwarrantedly harmed. In such scenarios, public trust in medicine may decline.^9,16^

In conclusion, Bostock’s paper is of historical interest and provides us an interesting glimpse into interwar Australian psychiatric practice. It also functions as a helpful reminder of the importance of remembering and carefully considering the complexities that the placebo effect brings to psychiatric research and clinical practice, now as well as then.
